# Development and validation of a risk prediction model for distant metastasis in muscle-invasive bladder cancer: a retrospective study integrating SEER data with external validation cohort and biomarker analysis

**DOI:** 10.3389/fonc.2025.1607173

**Published:** 2025-09-10

**Authors:** Quanqing Tang, Yutong Li, Kaifeng Liu, Gaozhen Huang, Liangmeng Gao, Yiqi Tang, Hongwei Liu

**Affiliations:** Laboratory of Urology, Affiliated Hospital of Guangdong Medical University, Zhanjiang, Guangdong, China

**Keywords:** nomogram, distant metastasis, bladder cancer, seer database, machine learning, biomarker

## Abstract

**Background:**

Bladder cancer (BCa) ranks among the most prevalent cancers in men, with a subset of patients developing distant metastases (DM), resulting in poor prognosis. This study aims to develop and validate a nomogram to predict DM in patients with BCa, utilizing machine learning techniques to identify potential biomarkers.

**Methods:**

Clinical data from patients with BCa diagnosed between January 2010 and December 2015 were retrospectively retrieved from the Surveillance, Epidemiology, and End Results (SEER) database and randomly split into a training cohort (n = 1,619) and an internal validation cohort (n = 694). An external validation cohort (n = 112) was obtained from the Affiliated Hospital of Guangdong Medical University between January 2021 and December 2023. Independent risk factors for DM were identified using univariate and multivariate logistic regression analyses and incorporated into the nomogram. Predictive accuracy was evaluated using calibration curves, and the nomogram's discriminative ability was compared with traditional staging systems by calculating the area under the curve (AUC).

**Results:**

Tumor size ≥ 3 cm, N stage (N1–N3), and lack of surgery were found to be independent risk factors for DM, all of which were included in the nomogram. ROC curve analysis demonstrated robust predictive performance, with AUC values of 0.732 in the training cohort, 0.750 in the internal validation cohort, and 0.968 in the external validation cohort. Additionally, calibration curves consistently showed good predictive accuracy across all cohorts. Machine learning methods, including LASSO and Random Forest, identified ADH1B as a potential biomarker for BCa, displaying exceptional diagnostic and prognostic performance (AUC = 0.983).

**Conclusion:**

This study, based on the SEER database and an external validation cohort, identified independent risk factors for DM in BCa and revealed ADH1B as a novel biomarker, offering new perspectives for clinical prediction and personalized treatment.

## Introduction

Bladder cancer (BCa) is among the most prevalent malignant tumors of the urothelial system, with increasing morbidity and mortality rates globally (1). According to the American Cancer Statistics Report, BCa ranks as the fourth most common malignancy in men (2). In 2024, there were 83,190 new cases of BCa worldwide, resulting in 16,840 deaths from the disease (3). BCa is categorized into non-muscle invasive bladder cancer (NMIBC) and muscle-invasive bladder cancer (MIBC) based on tumor invasion depth (4). Urothelial carcinoma (UC) is the predominant pathological type of BCa, comprising approximately 90% of cases, while squamous cell carcinoma (SCC) and adenocarcinoma each account for only 2%-3% (5). Risk factors such as smoking, prolonged exposure to industrial chemicals, and chronic bladder infections are associated with BCa pathogenesis (6). BCa often presents insidiously, with early-stage symptoms being subtle, and only 2%-5% of patients show microscopic hematuria (7). Lymphatic spread is one of the primary metastatic pathways for BCa (8), with common sites of distant metastasis (DM) including lymph nodes (25.4%), bone (24.7%), urethra (23.5%), lung (19.4%), liver (18.1%), and brain (3.1%) (9). Transurethral resection of bladder tumor (TURBT) is the standard treatment for NMIBC. However, 40%-80% of patients with NMIBC experience tumor recurrence, and 10%-25% develop metastases (10). Although the BCa is responsive to chemotherapy, prognosis worsens once DM occurs (11).

Clinical prediction models, also known as risk scores, are built on analyzing multiple etiological factors to construct statistical frameworks that estimate the likelihood of specific outcomes in populations with defined characteristics (12–14). Although the risk factors for DM in BCa have been explored, consensus on the exact determinants remains elusive. To address this gap, this study conducted research based on the SEER database and an external cohort.

Multi-omics technologies have generated extensive biological data, presenting both opportunities and challenges for analysis (15). Moreover, machine learning techniques have proven effective in mining omics data to identify prognostic biomarkers and elucidate disease mechanisms, contributing to biomarker discovery and enhancing our understanding of the biological underpinnings of various diseases (16). Biomarkers are also crucial for predicting cancer types, survival rates, and staging (17). The present study integrated statistical analysis with machine learning methods to enhance the stability and interpretability of biomarker identification in BCa.

Thus, this study aims to develop a predictive model for DM in patients with BCa using the SEER database. Clinical data from patients with BCa diagnosed between January 2021 and December 2023 were extracted from the SEER database, and a clinical prediction model was constructed using R software. To validate the model’s accuracy, clinical data from patients with BCa admitted to the Department of Urology at the Affiliated Hospital of Guangdong Medical University from January 2021 to December 2023 were employed. Additionally, potential biomarkers for BCa were identified by integrating multiple BCa datasets and applying various machine learning methods.

## Materials and methods

### Patients and inclusion criteria

Patient data were retrieved from the Surveillance, Epidemiology, and End Results (SEER) database, supported by the National Cancer Institute, as of May 1, 2023. Clinicopathological information was obtained using SEER*Stat software (version 8.4.3). The SEER-18 registries database (November 2022 Submission), covering approximately 28% of the U.S. population, was used. The study period spanned from January 2010 to December 2015, with the 2015 cutoff ensuring a minimum of 5 years of follow-up for all patients.

**Figure 1:**
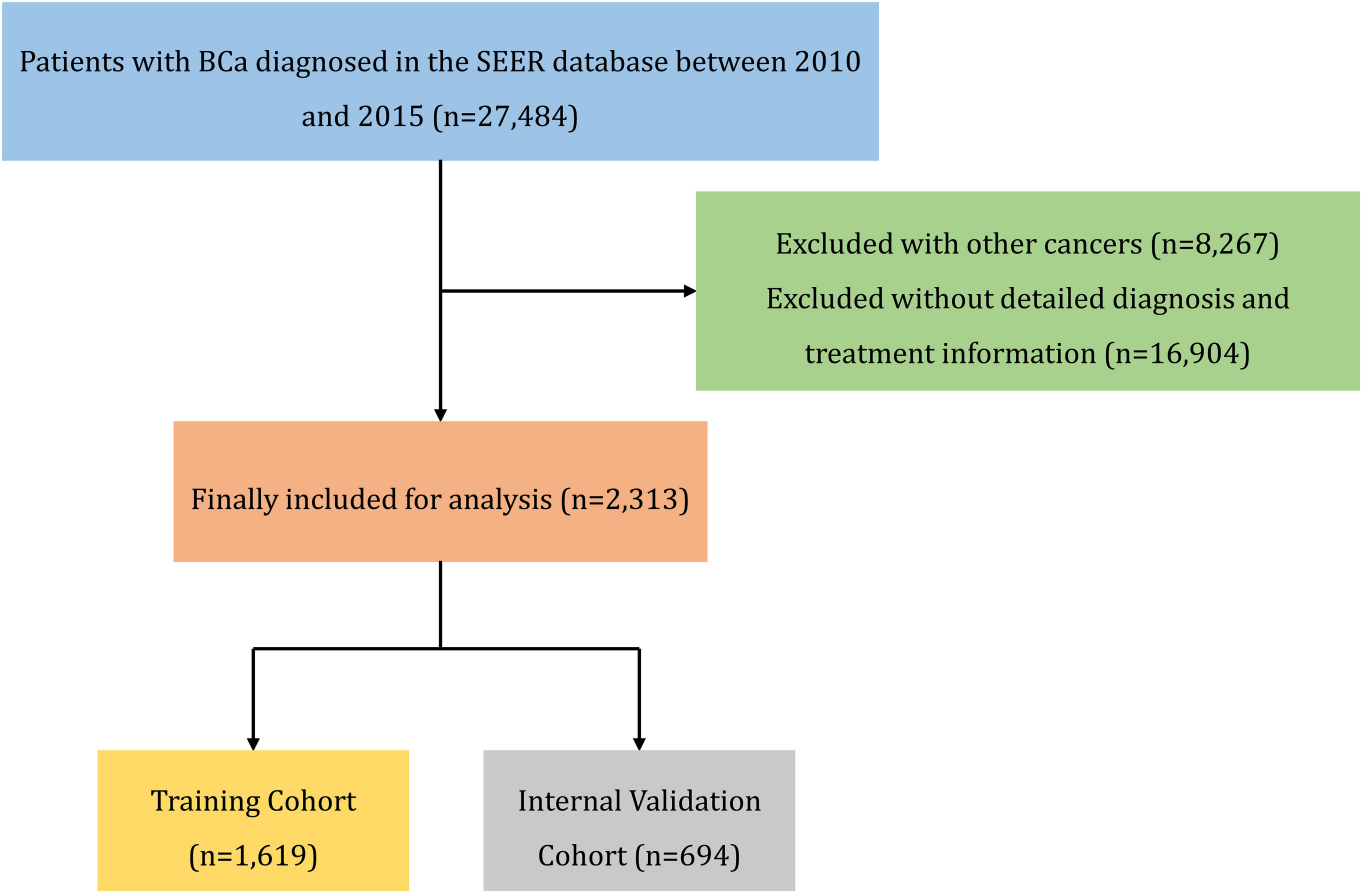
Specific flow chart of the SEER analysis.

Patients with BCa were selected based on the histological code from the International Classification of Diseases for Oncology, Third Edition (ICD-O-3). Inclusion criteria included: a) a pathological diagnosis of UC and b) BCa as the primary cancer. Exclusion criteria were: a) diagnoses outside the study period, b) incomplete basic information (e.g., surgery, chemotherapy, tumor size, metastasis), and c) the presence of other tumors. The SEER data used in this study are publicly available and exempt from Ethics Committee approval. The patient selection flowchart is shown in **Figure 1**.

Additionally, demographic data and clinical characteristics of patients with BCa admitted to the Department of Urology at the Affiliated Hospital of Guangdong Medical University from January 2021 to December 2023 were collected. The inclusion and exclusion criteria for the Chinese cohort mirrored those applied to the SEER cohort. Informed consent was obtained from all participants. This retrospective study adhered to the Declaration of Helsinki and was approved by the Clinical Research Ethics Committee of the Affiliated Hospital of Guangdong Medical University (ethical approval number: PJKT2024-105).

### Study factors

This study incorporated factors such as age, gender, race, tumor size, tumor grade, T stage, N stage, M stage, radiotherapy status, chemotherapy status, and surgery. To ensure cross-database compatibility, all variables were harmonized between SEER and the external cohort using standardized coding. Tumor size was categorized as < 3 cm or ≥ 3 cm based on preoperative imaging (CT/MRI) or pathological reports.

Treatment variables (surgery, chemotherapy, and radiotherapy) were classified as “Yes” if any modality was administered, regardless of specific regimens, due to limitations in SEER data. Metastasis status (M stage) was confirmed *via* imaging (CT/MRI/PET-CT) or biopsy according to AJCC 8^th^ edition criteria. Data processing was standardized: continuous variables (e.g., age) were categorized before analysis, and missing data led to exclusion based on the inclusion criteria. Bone, brain, and lung metastases were used as the primary outcome factors.

### Construction and validation of nomograms

Continuous variables were converted into categorical variables for analysis. For nomogram construction, enrolled patients were randomly divided into a training group and a validation group in a 7:3 ratio. Univariate logistic regression analysis was performed to identify risk factors for predicting DM in patients with BCa. Factors with a *P*-value < 0.05 from the univariate analysis were further assessed using multivariate logistic regression to identify independent risk factors. These independent risk factors were then used to develop a predictive model for DM risk in patients with BCa using R software, with the results visualized in a nomogram. A calibration curve was generated to depict the relationship between actual and predicted probabilities. Additionally, the receiver operating characteristic (ROC) curve was plotted, and the area under the curve (AUC) was calculated to evaluate the model’s prediction accuracy. An independent external validation cohort was used to calibrate and validate the prediction model.

### Comparison with published research

PubMed was searched for studies on BCa utilizing the SEER database to investigate DM. The final outcomes and predictive models from these studies were extracted for comparison with the current research. Six relevant studies were identified.

### BCa transcriptome data acquisition

Four BCa transcriptomic datasets (GSE13507, GSE37817, GSE166716, GSE256292) were obtained from the GEO database (https://www.ncbi.nlm.nih.gov/geo/). After batch effect removal and data normalization, these datasets were used as the training cohort. Additionally, the latest TCGA-BCa transcriptomic data from the TCGA-BLCA database (https://portal.gdc.cancer.gov/) was acquired to serve as the validation cohort.

### Constructing a machine learning model to screen BCa biomarkers

To identify robust biomarkers for BCa prognosis, a comprehensive computational pipeline was developed, integrating multiple transcriptomic datasets and advanced machine learning. Rigorous preprocessing and harmonization of all datasets ensured data quality and comparability. For microarray-based GEO datasets, background correction and quantile normalization were performed using the Robust Multi-array Average (RMA) algorithm in R. Batch effects across different studies and platforms were corrected using the ComBat algorithm from the sva package. RNA-seq data from TCGA were processed through a standardized pipeline, which included quality control with FastQC, adapter trimming with Trim Galore, and alignment to the GRCh38 reference genome with STAR. Gene-level counts were generated using featureCounts and normalized via the DESeq2 variance stabilizing transformation. Quality control was verified through PCA, which demonstrated that the corrected biological factors accounted for over 80% of the variance.

For feature selection, LASSO regression was applied via the glmnet package, analyzing all 18,543 genes from batch-corrected data against binary metastasis status using 10-fold cross-validation to determine the optimal λ parameter at 1 standard error from the minimum mean squared error. Genes with non-zero coefficients at this threshold were considered candidate biomarkers, and their stability was validated through 100 bootstrap iterations. Only genes selected in more than 80% of the iterations were advanced.

### Potential biomarker validation and functional analysis

The final validation incorporated a random forest implementation via the ranger package, with 1,000 trees and a node size of 5, ranking features by permutation-based importance scores. The integration framework prioritized consensus genes that appeared in multiple algorithm outputs, requiring consistent directional effects and validation in the independent TCGA cohort with AUC > 0.75. Biological relevance was further assessed through KEGG pathway analysis, protein-protein interaction networks, and literature verification of cancer associations.

### Statistical analysis

Statistical processing was conducted using R software. Count data were expressed as numbers or percentages, while continuous variables following a normal distribution were presented as mean ± standard deviation. Categorical variables were expressed as percentages. Univariate and multivariate logistic regression analyses were performed on SEER database data to identify significant risk factors (*P* < 0.05). These risk factors were used to construct a predictive model for DM in patients with BCa. The model’s calibration curve, ROC curve, and AUC were generated using Hmisc and ROCR packages in R.

For external validation, the identical nomogram scoring algorithm from the SEER training cohort was applied to the external cohort without recalibration. Model performance, including AUC and calibration curves, was evaluated consistently across all cohorts to eliminate methodological bias. A *P*-value < 0.05 was considered statistically significant.

## Results

### Patient characteristics

A total of 27,484 patients with BCa from January 2010 to December 2015 were sourced from the SEER database for this study. After excluding 8,267 patients with other cancers and 16,904 patients with incomplete information, 2,313 eligible cases were selected. These cases were randomly divided into a training cohort of 1,619 cases and an internal validation cohort of 694 cases. The external validation cohort data (n = 112) were sourced from the Affiliated Hospital of Guangdong Medical University, covering the period from January 2021 to December 2023, based on the same inclusion and exclusion criteria.

No statistically significant differences were observed in the demographic and clinical characteristics between the training and internal validation cohorts, as shown in **Table 1**. The majority of patients were aged 65 years or older, comprising 61.05% of the sample. In terms of T stage, patients with T2-T4 tumors accounted for 94.16%, while those with Tis, Ta, or T1 tumors made up only 5.84%. The training cohort included 63 patients diagnosed with DM, and the internal validation cohort had 27 such patients. A substantial proportion of patients underwent surgery (99.78%), and a significant portion received chemotherapy (52.75%).

**Table 1:** Demographic and pathological characteristics of patients with BCa.

Variable	Training cohort	Internal validation cohort	External validation cohort	*χ* ^2^	*P*-value
	(n = 1619) (%)	(n = 694) (%)	(n = 112) (%)		
Age				0.577	0.448
< 65 years	622 (38.42)	279 (40.20)	15 (13.39)		
≥ 65 years	997 (61.58)	415 (59.80)	97 (86.61)		
Sex				0.001	0.977
Female	422 (26.07)	182 (26.22)	28 (25.00)		
Male	1197 (73.93)	512 (73.78)	84 (75.00)		
Race				0.077	0.962
Black	124 (7.66)	51 (7.35)	0 (0.00)		
White	1405 (86.78)	605 (87.17)	0 (0.00)		
Others	90 (5.56)	38 (5.48)	112 (100.00)		
Tumor size				0.399	0.139
<3 cm	480 (30.09)	196 (26.34)	75 (66.96)		
≥3 cm	1139 (69.91)	498 (73.66)	37 (30.04)		
Grade				5.496	0.154
Well	9 (0.56)	2 (0.29)	4(3.58)		
Moderate	30 (1.85)	12 (1.73)	15(13.39)		
Poor	345 (21.31)	177 (25.50)	39(34.82)		
Anaplastic	1235 (76.28)	503 (72.48)	54(48.21)		
T stage				0.037	0.847
Tis Ta T1	93 (5.74)	42 (6.05)	71 (63.39)		
T2-T4	1526 (94.26)	652 (93.95)	41 (36.61)		
N stage				2.921	0.404
N0	1029 (63.56)	460 (66.28)	53 (47.32)		
N1	241 (14.88)	89 (12.83)	28 (25.00)		
N2	300 (18.53)	120 (17.29)	17 (15.18)		
N3	49 (3.03)	25 (3.60)	14 (12.50)		
M stage				2.274	1.000
M0	1556 (96.11)	667 (96.11)	92 (82.14)		
M1	63 (3.89)	27 (3.89)	20 (17.86)		
Radiotherapy				1.190	0.275
No	1540 (95.12)	668 (96.25)	88 (78.57)		
Yes	79 (4.88)	26 (3.75)	24 (21.43)		
Chemotherapy				1.102	0.294
No	753 (46.51)	340 (48.99)	35 (31.25)		
Yes	866 (53.49)	354 (51.01)	77 (68.75)		
Surgery				4.459	1.000
No	4 (0.25)	1 (0.14)	34 (30.36)		
Yes	1615 (99.75)	693 (99.86)	78 (69.64)		

In the external validation cohort, 15 cases (13.39%) were in the < 65 years group, and 97 cases (86.61%) were in the ≥ 65 years group. Regarding T stage, 71 cases (63.39%) were in the Tis, Ta, T1 group, while 41 cases (36.61%) were in the T2-T4 group. Of the cohort, 35 cases (31.25%) did not undergo chemotherapy, while 77 cases (68.75%) received chemotherapy. In terms of surgery, 34 cases (30.36%) did not undergo surgery, and 78 cases (69.64%) did.

### Univariate and multivariate analyses

**Figure 2:**
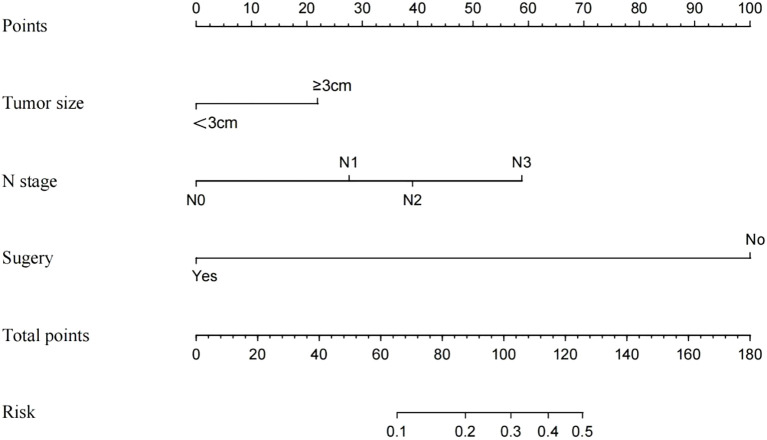
Nomogram prediction model of BCa distant metastasis.

Univariate and multivariate logistic regression analyses were performed on the training cohort to assess each prognostic factor, as shown in **Table 2**. The univariate analysis identified grade, T stage, N stage, radiotherapy, chemotherapy, and surgery as significant factors (*P* < 0.05). In the multivariate regression analysis, a maximum tumor diameter of ≥ 3 cm, N1-N3 stage, and the absence of surgery (*P* < 0.05) emerged as independent prognostic factors. These factors were subsequently included in the nomogram, as depicted in **Figure 2**.

**Table 2:** Univariate and multivariate analyses of the clinicopathological parameters using the SEER training cohort.

Variable	Univariate			Multivariate		
	HR	95%*CI*	*P*	HR	95%*CI*	*P*
Age						
<65 years	Reference					
≥65 years	1.014	0.660 - 1.581	0.957			
Sex						
Female	Reference					
Male	0.809	0.516 - 1.303	0.451			
Race						
Black	Reference					
White	0.771	0.394 - 1.714	0.556			
Others	0.915	0.290 - 2.678	0.893			
Tumor size						
< 3 cm	Reference			Reference		
≥ 3 cm	2.605	1.483 - 4.966	0.009	2.165	1.218 - 4.168	0.037
Grade						
Well	Reference					
Moderate	2.783	2.504 - 4.142	0.052			
Poor	5.592	4.871 - 6.158	0.006			
Anaplastic	4.311	3.197 - 6.231	0.034			
T stage						
Tis Ta T1	Reference					
T2-T4	3.896	1.031 - 37.331	0.180			
N stage						
N0	Reference			Reference		
N1	2.644	1.217 - 4.551	0.009	2.534	1.326 - 4.716	0.015
N2	4.387	3.001 - 8.417	<0.001	3.819	2.239 - 6.566	<0.001
N3	8.408	2.382 - 13.770	<0.001	8.145	3.551 - 17.447	<0.001
Radiotherapy						
No	Reference			Reference		
Yes	2.576	1.220 - 4.906	0.024	1.701	0.760 - 3.412	0.240
Chemotherapy						
No	Reference			Reference		
Yes	1.777	1.146 - 2.813	0.035	1.217	0.765 - 1.970	0.494
Surgery						
No	Reference			Reference		
Yes	0.039	0.007 - 0.226	0.001	0.028	0.004 - 0.196	0.002

### Nomograms construction and validation

Three independent risk factors—maximum tumor diameter ≥ 3 cm, N1-N3 stage, and absence of surgery—identified through logistic regression analysis were used as the final predictors in this study. A clinical prediction model, developed using R software, was presented as a nomogram to predict DM in patients with BCa (**Figure 2**). Surgery had the most significant contribution to prognosis, followed by N stage and tumor size. In the nomogram, each factor is aligned with a fractional axis, and a vertical line is drawn to obtain the corresponding score. The total score is derived by summing the scores of each factor on the total axis, reflecting its influence on the progression of DM in BCa.

**Figure 3:**
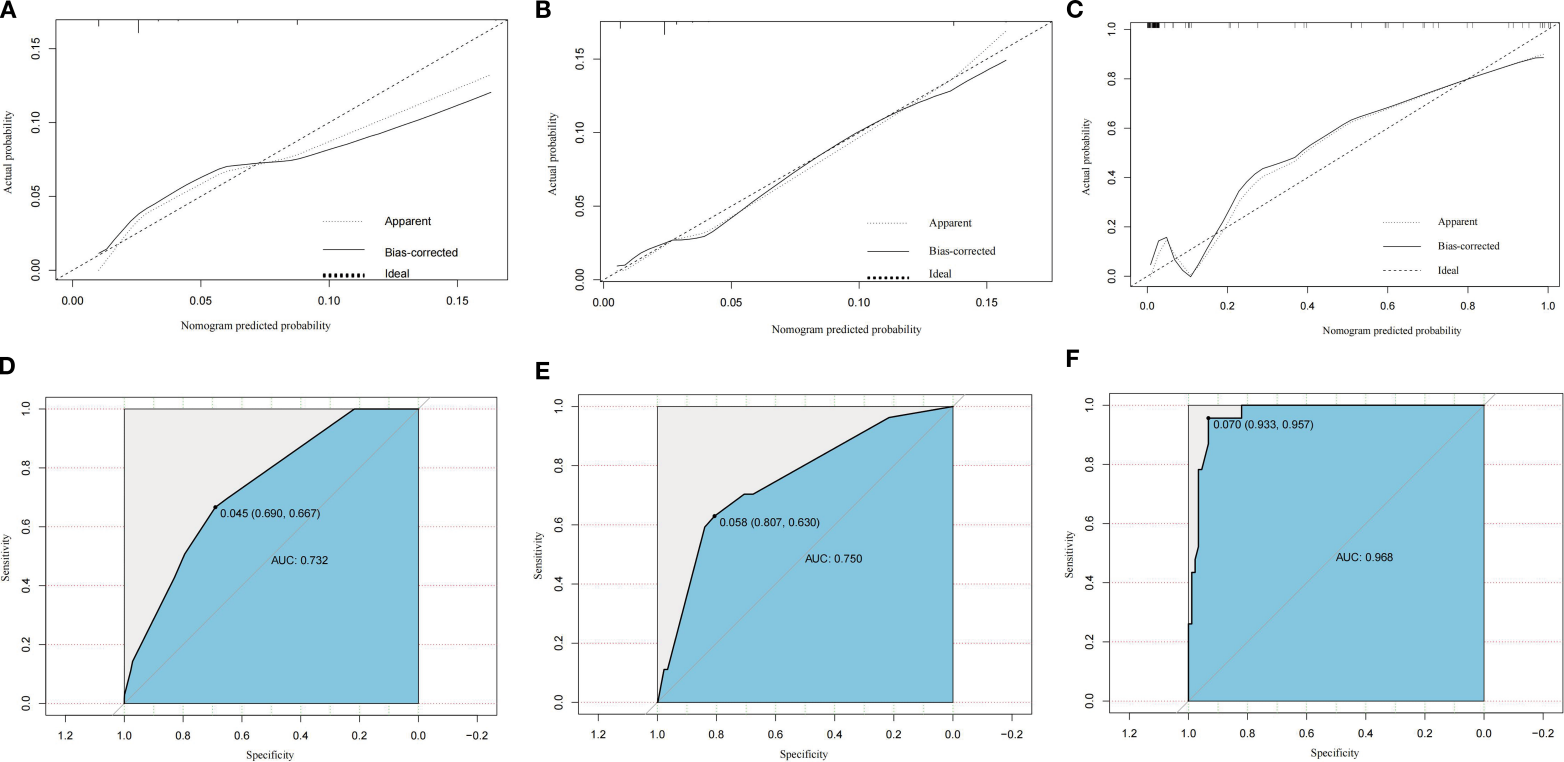
Calibration plots of the constructed nomogram for predicting distant metastasis risk in the training cohort **(A)**, internal validation cohort **(B)**, and external validation cohort **(C)**. Receiver operating characteristic (ROC) curves for discrimination of distant metastasis risk in the training cohort **(D)**, internal validation cohort **(E)**, and external validation cohort **(F)**. AUC, area under the curve; SEER, Surveillance, Epidemiology, and End Results.

ROC curve analysis showed that the optimal diagnostic cut-off point for the training group was 0.045, with a sensitivity of 66.70% and specificity of 69.00%. For the internal validation group, the cut-off point was 0.058, with sensitivity at 63.00% and specificity at 80.70%. The external validation group showed a cut-off point of 0.070, with sensitivity and specificity at 95.70% and 93.30%, respectively. Calibration curve and standard curve fitting for the training group were moderate, with an AUC of 0.732, indicating strong discriminative ability of the prediction model. The predicted calibration curve for the internal validation group closely matched the standard curve, indicating the nomogram’s robust predictive capacity. The AUC for the internal validation group was 0.750, and for the external validation group, it was 0.968 (**Figure 3**).

### Analysis of external validation performance

The external validation cohort demonstrated a higher AUC (0.968) compared to the training (0.732) and internal validation (0.750) cohorts. This discrepancy is likely due to the distinct clinicopathological profile of the external cohort (**Table 1**), which included a significantly larger proportion of non-muscle invasive tumors (Tis/Ta/T1: 63.39% vs. 5.84% in SEER), a higher rate of DM (M1: 17.86% vs. 3.89% in SEER), and a substantially lower surgery rate (non-surgical: 30.36% vs. 0.25% in SEER).

Given that the absence of surgery and advanced N stage were the strongest predictors in our nomogram, the higher concentration of these high-risk features in the external cohort likely enhanced the model’s discriminative power. Although the sample size was limited (n = 112), the rigorous inclusion criteria ensured data quality. These differences reflect real-world patient allocation and provide a robust test of the model’s performance across diverse settings.

### Comparing multiple research models

The predictive performance of our model for DM in BCa was compared with several recently published models developed using SEER data. As summarized in **Table 3**, the internally validated C-index for our model, incorporating the predictors tumor size ≥ 3 cm, nodal stage N1-N3, and non-receipt of surgery, was 0.750. This performance is comparable to, or exceeds, the predictive accuracy of several SEER-based studies on BCa DM, which reported C-indices/AUCs ranging from 0.688 to 0.722 (**Table 3**).

**Table 3:** Comparison of the predictive power of exposure factors for BCa DM between this study and other studies.

Research	Outcomes	Exposure	C-index/AUC	PMID
This research (internal validation group)	BCa With DM	T ≥ 3 cm, N1-N3, not undergo surgery	0.750	/
This research (external validation group)	BCa With DM	T ≥ 3 cm, N1-N3, not undergo surgery	0.968	/
Liangjun Tao, et al.	BCa With DM	Marital Status	0.722	33194738
Xin Chang Zou, et al.	BCa With DM	non-regional lymph nodes, age, and chemotherapy	0.877	39735606
Jiafeng Shou, et al.	BCa With DM	different distant metastases pattern	/	34028594
Ping Wang, et al.	BCa With DM	significance of histology type and metastatic pattern	0.711	33107706
Qian Deng, et al.	BCa With DM	T2-T4 MIBC patients aged > 18 years old	0.688	40189676
Jiaxiang Ji, et al.	BCa With DM	efficacy of cystectomy	/	39974803

Notably, when externally validated using our independent, prospectively collected cohort, the predictive power of our model significantly improved, achieving an outstanding C-index of 0.968. This result far surpasses the performance metrics of all other SEER-based studies, including the highest previously reported AUC of 0.877. This comparative analysis highlights the robust discriminatory power of our model, especially its exceptional performance in the external validation cohort, suggesting strong generalizability beyond the derivation cohort.

### Machine learning explores potential biomarkers of BCa

**Figure 4:**
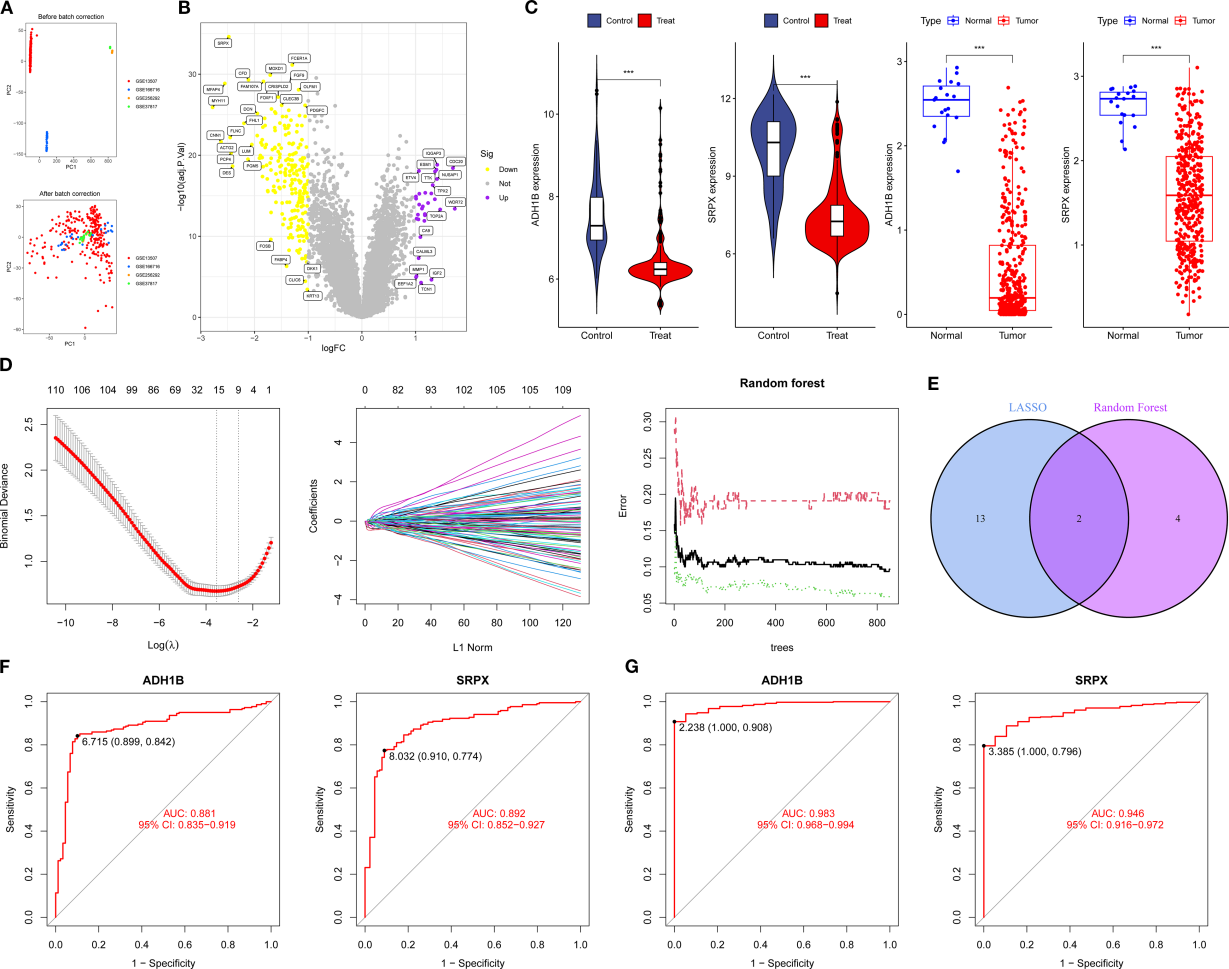
Machine learning screening for potential biomarkers of BCa: **(A, B)** PCA and BCa volcano plots of differentially expressed genes. **(C)** Expression levels of SRPX and ADH1B. **(D)** LASSO and Random Forest analysis. **(E)** Intersection gene Venn plot. **(F, G)** ROC curves for the training and validation groups. *P < 0.05, **P < 0.01, ***P < 0.001.

PCA demonstrated excellent data quality across datasets, with corrected biological factors explaining more than 80% of the variance (**Figure 4A**). Batch effect analysis identified 253 differentially expressed genes in BCa that met the criteria of logFC > 1 and *P* < 0.05 (**Figure 4B**). Integrated machine learning approaches, including LASSO regression and Random Forest algorithms, identified 15 and 6 potential BCa biomarkers, respectively, with SRPX and ADH1B emerging as consensus candidates from both methods (**Figures 4D, E**). Comparative expression analysis revealed that both SRPX and ADH1B were significantly downregulated in tumor tissues compared to normal bladder tissues (*P* < 0.05) across both training and validation cohorts, suggesting their potential protective roles in BCa pathogenesis (**Figure 4C**). ROC curve analysis demonstrated robust diagnostic performance, with AUC values exceeding 0.7 for both biomarkers in all cohorts (**Figures 4F, G**). These findings position SRPX and ADH1B as promising diagnostic biomarkers with potential clinical utility for BCa prognosis prediction. Notably, ADH1B exhibited particularly strong performance in the validation cohort (AUC = 0.983 in the TCGA dataset), highlighting its potential as a superior biomarker candidate.

**Figure 5:**
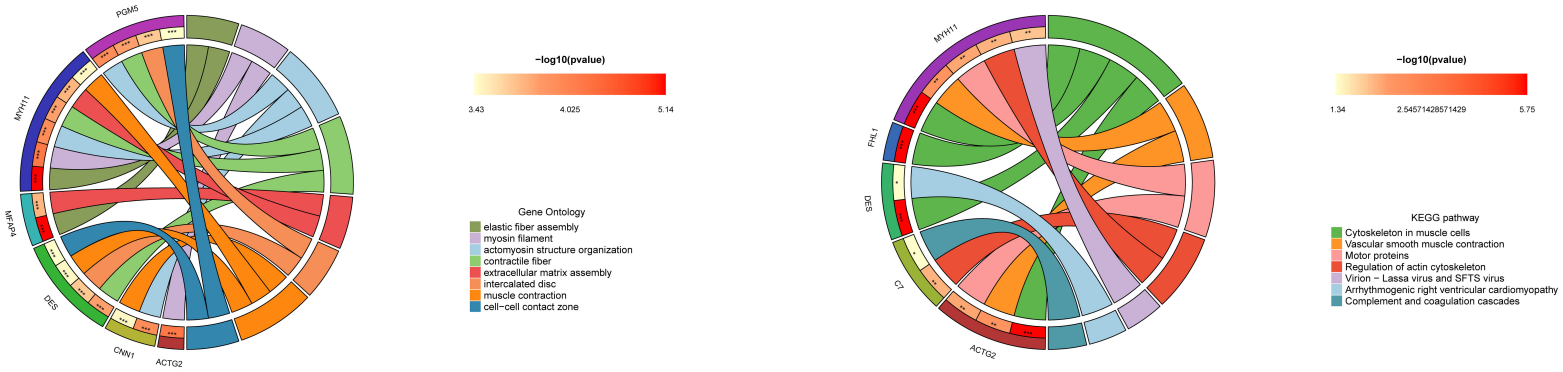
Functional analysis of GO and KEGG mediated by ADH1B in BCa.

GO enrichment analysis of ADH1B-associated genes in BCa revealed significant functional associations across biological processes, cellular components, and molecular functions. The most prominent findings were centered around muscle system-related activities, with strong enrichment for muscle contraction, actomyosin structure organization, and smooth muscle contraction (**Figure 5**). Cellular component analysis showed notable associations with contractile fibers, myosin filaments, and focal adhesions, while molecular function analysis revealed ADH1B’s involvement in calmodulin binding and actin binding. ADH1B may influence BCa progression through modulation of muscle-related functions and extracellular matrix organization, potentially affecting tumor stiffness and metastatic potential.

KEGG pathway enrichment analysis of ADH1B-associated genes revealed significant involvement in several key biological pathways related to the BCa progression. The most significantly enriched pathway was cytoskeletal organization in muscle cells, with four genes (FHL1, MYH11, DES, ACTG2) showing strong associations. Vascular smooth muscle contraction and motor protein function also demonstrated significant enrichment, further supporting ADH1B’s potential role in muscle-related cellular processes (**Figure 5**). Additionally, the regulation of the actin cytoskeleton pathway was notably represented, suggesting that ADH1B may influence cancer cell motility and metastasis.

## Discussion

BCa ranks as the second most common malignancy within the urothelial system (18). At the time of diagnosis, 10%-15% of patients typically present with disease progression or metastasis (19). For patients with BCa without DM, surgical intervention is the preferred first-line treatment. In contrast, patients with DM are more commonly managed with chemotherapy (20–22), immunotherapy (23), or targeted therapy (24), commonly recommended (25). Despite aggressive surgical resection, over 50% of patients with BCa will develop distant micro-metastases postoperatively (26). Patients with DM from BCa can still benefit from interventions following the resection of both metastases and primary tumors (27). Platinum-based chemotherapy remains the cornerstone of treatment for metastatic UC (mUC), but the median overall survival (mOS) rarely exceeds 3 to 6 months (28). While surgical and chemotherapeutic approaches can manage BCa effectively and improve OS rates (29), the high recurrence and metastasis rates post-surgery contribute to a 5-year survival rate of only 50%-60% for patients with BCa (19).

Currently, the diagnosis of tumor-derived DM primarily depends on computed tomography (CT), magnetic resonance imaging (MRI), and positron emission tomography (PET). While PET offers superior sensitivity compared to CT and MRI, its high cost restricts its routine clinical use (30). Thus, there is a pressing need for the development of novel diagnostic methodologies capable of more sensitively and effectively identifying high-risk individuals for DM prior to surgery. The nomogram, a multivariate prediction model based on individual patient characteristics, serves as a valuable tool. It not only estimates disease risk but also assists clinicians in identifying high-risk groups and tailoring more appropriate treatment strategies (31).

Among the patients in the SEER database, 2,223 (96.11%) did not have DM, while 90 (3.89%) were diagnosed with DM. In the external validation group, 92 patients (82.14%) had no DM, and 20 patients (17.86%) had DM. Ten factors were evaluated: age, sex, race, tumor size, grade, T stage, N stage, radiotherapy, chemotherapy, and surgery. Univariate logistic regression revealed that tumor size, N stage, radiotherapy, chemotherapy, and surgery significantly influenced DM in patients with BCa. Multivariate logistic regression further identified the maximum tumor diameter of ≥ 3 cm, N1-N3 stage, and absence of surgical intervention as independent risk factors for DM in BCa. A nomogram prediction model was then developed based on these three independent risk factors. Model’s predictive performance was assessed, with the calibration and standard curves of the training group aligning well, yielding an AUC of 0.732, indicating good discriminative ability. The calibration curve for the internal validation group closely matched the standard curve, with an AUC of 0.750, demonstrating the model’s strong predictive capability. The external validation group achieved an AUC of 0.968. The prediction model developed in this study outperforms those based on single factors, aiding clinicians in promptly and accurately identifying high-risk patients with BCa and informing clinical intervention strategies.

Epidemiological studies of BCa indicate a predominant impact on elderly male patients, with a median age of diagnosis of 70 years (32–34) and a male-to-female ratio of approximately 77% to 23% (35). Research supports the higher incidence in men compared to women (32). Additionally, a study demonstrated that age and pathological grade influence the progression of NMIBC (34). Another study identified age and gender, particularly women aged 40–60 years, as independent risk factors for DM in BCa (27).

In this study, the training and internal validation groups consisted of 901 patients (38.95%) under 65 years and 1,412 patients (61.05%) aged 65 years or older. The male-to-female ratio was approximately 74% male to 26% female, consistent with the existing literature. The external validation group reflected similar proportions. However, univariate logistic regression analysis did not find age to be a significant factor for DM in BCa, possibly due to the classification of age groups in this study. More detailed age stratification may be required to fully understand its impact.

The data from this study identified a maximum tumor diameter ≥ 3 cm and N1-N3 stage as independent risk factors for DM in patients with BCa. Interestingly, tumor stage and tumor grade did not significantly influence DM in BCa in the univariate logistic regression analysis, despite other studies demonstrating associations between these factors and DM in patients with BCa (36, 37), including lymph node infiltration and TNM stage. In a SEER database analysis, Shou et al. also found that BCa individuals with advanced tumor stages, positive lymph node metastasis, and high histological grades were susceptible to DM (38). As tumor stage advances, the degree of tumor cell differentiation decreases, weakening the adhesion between tumor cells and promoting metastasis. Furthermore, T stage, tumor grade, and tumor size significantly influence lymph node metastasis in BCa (39). Additional research has confirmed that higher T and N stages, as well as lower tumor differentiation, are independent risk factors for DM in BCa (40).

Surgical resection remains a critical aspect of BCa treatment. Several clinical studies have shown that transurethral resection can reduce the risk of recurrence and DM in patients with T2 BCa (41). The radical resection decreases recurrence and metastasis, thereby improving the survival rate of patients with BCa (42). The nomogram prediction model in this study suggests that patients who undergo surgery have a lower risk of DM compared to those who do not, which is consistent with findings from related studies. However, due to the lack of specific surgical methods and non-surgical data in the SEER database, patients were categorized solely based on whether they had surgery. Notably, some patients who did not undergo surgery may have been ineligible due to DM at the time of treatment, rather than opting out of surgery as early-stage patients. Univariate logistic regression analysis in this study revealed significant differences in the effects of radiotherapy and chemotherapy on DM in patients with BCa. One study reported that arterial perfusion combined with bladder perfusion chemotherapy can slow the progression of MIBC, reduce the recurrence rate of postoperative metastasis, and improve patients’ quality of life (43). Radiotherapy, a non-invasive treatment, has been shown to decrease the likelihood of postoperative recurrence and metastasis in patients with BCa (44). Additionally, radiotherapy serves as a palliative treatment for BCa individuals with DM (45), with improvements in pain typically observed within 2–6 weeks post-treatment (40).

Through comprehensive screening using the LASSO and Random Forest algorithms, SRPX and ADH1B were identified as potential biomarkers for BCa. Subsequent ROC curve and Kaplan-Meier survival analyses consistently demonstrated that ADH1B is the most promising novel biomarker for BCa, exhibiting exceptional diagnostic performance (AUC = 0.983 in the TCGA cohort, AUC = 0.881 in the GEO cohort) and significant prognostic value (*P* < 0.05). The protein encoded by ADH1B is a member of the alcohol dehydrogenase family, characterized by high ethanol oxidation activity and involvement in ethanol metabolism (46). In pan-cancer tissues, ADH1B expression is significantly downregulated (47). ADH1B activity levels in the serum of patients with BCa are significantly elevated, with higher activity potentially linked to metastatic tumors (48). A study by Masaoka et al. revealed that ADH1B is significantly associated with an increased BCa risk, with individuals carrying the ADH1B Arg+ variant having the highest risk of developing the disease (49). However, the molecular mechanisms through which ADH1B regulates and mediates BCa progression remain unclear. Our study demonstrates that ADH1B is downregulated in BCa tissues, where it functions as a protective factor. KEGG pathway analysis suggests that ADH1B primarily regulates complement and coagulation cascades, actin cytoskeleton organization, and muscle cell cytoskeletal functions. Nonetheless, its precise biological roles warrant further exploration through *in vitro* functional studies.

Although the prediction model developed in this study shows strong predictive capability, several limitations must be acknowledged. Firstly, this model relies on the SEER database, which lacks specific clinical details such as chemotherapy regimens, laboratory indicators, surgical methods, and critically, patient behavioral factors like smoking status and history. The absence of smoking data, a known risk factor for BCa progression, limits the ability to fully assess metastatic risk profiles. Additionally, the tumor size thresholds used in the model were derived from prior studies, which may not fully align with current clinical data, potentially introducing selection bias. As with any large-scale registry database, retrospective analyses using SEER data are subject to inherent limitations in data granularity and the potential for unmeasured confounding factors.

Secondly, while external validation was conducted, it relied on a cohort from a single institution with a relatively limited sample size (n = 112). The SEER training data covered the period from January 2010 to December 2015, whereas the external validation cohort comprised patients with BCa treated at the Affiliated Hospital of Guangdong Medical University between January 2021 and December 2023. The combination of a single-center validation cohort and the temporal gap between datasets raises concerns about the model’s generalizability. Variations in race, regional healthcare practices, diagnostic methodologies, and evolving treatment strategies over time may influence the model’s performance across different populations and contemporary clinical settings, highlighting the need for further validation.

Thirdly, to enhance broader applicability and robustness, future efforts should focus on expanding the dataset and incorporating validation across multiple regional hospitals. The model’s performance could also be enhanced by integrating additional clinically relevant variables, such as smoking history and detailed molecular markers, where available.

In summary, this study analyzed clinical data from 2,313 patients with BCa in the SEER database and 112 patients with BCa from the Affiliated Hospital of Guangdong Medical University. The study identified tumor size, N stage, and surgery as independent risk factors for DM in patients with BCa. The resulting nomogram prediction model demonstrated strong predictive performance, outperforming models based on single factors. To enhance the clinical applicability of the prediction model, future work should address the identified limitations and focus on improving relevant clinical indicators. Additionally, our research suggests that ADH1B may serve as a novel biomarker for BCa, exhibiting high sensitivity for its diagnosis.

## Data Availability

The raw data supporting the conclusions of this article will be made available by the authors, without undue reservation.
